# Retrospective study of the epidemiological risk and serological diagnosis of human babesiosis in Asturias, Northwestern Spain

**DOI:** 10.1186/s13071-023-05817-x

**Published:** 2023-06-09

**Authors:** Estrella Montero, María Folgueras, Mercedes Rodriguez-Pérez, Laura Pérez-ls, Javier Díaz-Arias, Maria Meana, Belén Revuelta, Karita Haapasalo, Julio Collazos, Víctor Asensi, Luis Miguel Gonzalez

**Affiliations:** 1 Parasitology Reference and Research Laboratory, Centro Nacional de Microbiología, Instituto de Salud Carlos III, Majadahonda, 28220 Madrid, Spain; 2 Infectious Diseases Unit, Hospital Universitario Central de Asturias, Oviedo University School of Medicine, Oviedo, Spain; 3 Microbiology Service, Hospital Universitario Central de Asturias, Oviedo University School of Medicine, Oviedo, Spain; 4 Researcher, Group of Translational Research in Infectious Diseases, Instituto de Investigación Sanitaria del Principado de Asturias (ISPA), Oviedo, Spain; 5 Internal Medicine, Hospital Alvarez-Buylla, Mieres, Asturias Spain; 6 Department of Bacteriology and Immunology, Medicum, University of Helsinki, 00014 Helsinki, Finland; 7 Infectious Diseases Unit, Hospital de Galdácano, Vizcaya, Spain; 8 Infectious Diseases Unit, Hospital Universitario Central de Asturias and Group of Translational Research in Infectious Diseases, Instituto de Investigación Sanitaria del Principado de Asturias (ISPA), Oviedo, Spain

**Keywords:** *Babesia divergens*, Epidemiological risk, Serological diagnosis, Ticks, Vector-borne diseases

## Abstract

**Background:**

Babesiosis is a globally growing tick-borne disease in humans. Severe babesiosis caused by *Babesia divergens* has been reported in two patients from Asturias (Northwestern Spain), suggesting an undetected risk for the disease. To analyze this risk, we retrospectively evaluated the seroprevalence of babesiosis in the Asturian population from 2015 through 2017, a period covering the intermediate years in which these two severe cases occurred.

**Methods:**

Indirect fluorescent assay (IFA) and Western blot (WB) were performed to detect *B. divergens* IgG antibodies in 120 serum samples from Asturian patients infected with the tick-transmitted spirochete *Borrelia burgdorferi* sensu lato, a condition that indicates exposure to tick bites.

**Results:**

This retrospective study confirmed a *B. divergens* seroprevalence rate of 39.2% according to IFA results. *B. divergens* incidence was 7.14 cases/100,000 population, exceeding previously reported seroprevalence rates. No differences in epidemiology and risk factors were found between patients infected solely with *B. burgdorferi* s.l. and those infected with *B. burgdorferi* s.l. and with IgG antibodies against *B. divergens*. This last group of patients lived in Central Asturias, had a milder clinical course and, according to WB results, developed different humoral responses against *B. divergens*.

**Conclusions:**

*Babesia divergens* parasites have circulated for several years in Asturias. Epidemiological evidence of babesiosis makes Asturias an emerging risk area for this zoonosis. Human babesiosis could also be relevant in other Spanish and European regions affected by borreliosis. Hence, the potential risk of babesiosis on human health in Asturias and other European forest regions needs to be addressed by the health authorities.

**Graphical Abstract:**

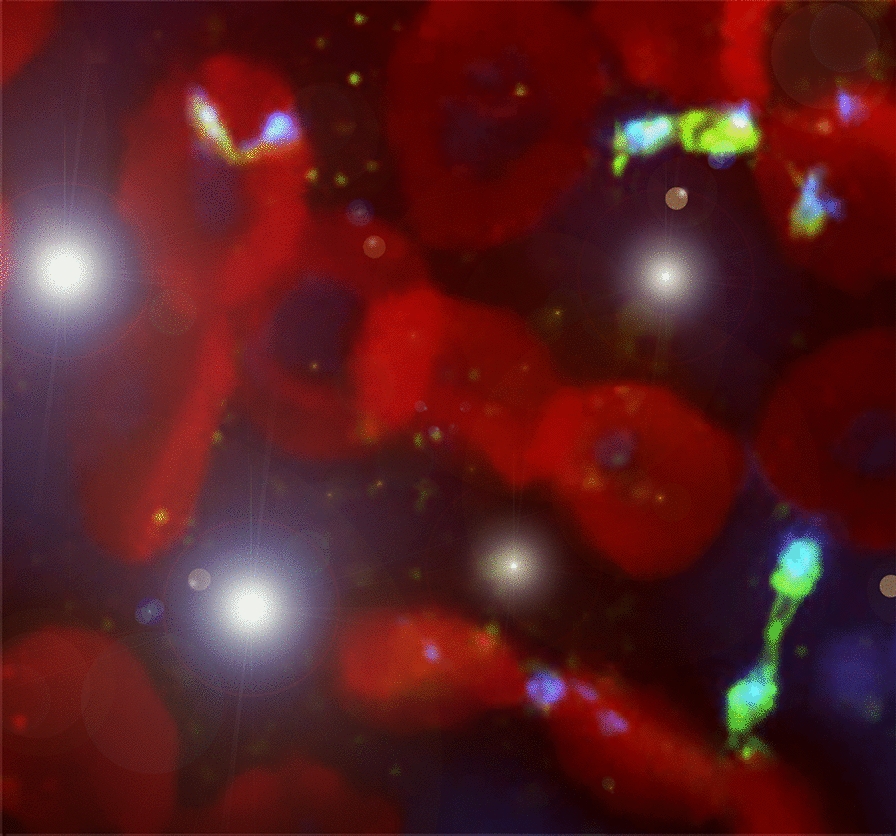

## Background

*Babesiosis* is an emerging tick-borne disease caused by apicomplexan parasites of the genus *Babesia* [1].

*Babesia divergens* is the primary agent responsible for babesiosis in humans and cattle in Europe [2, 3]. This intraerythrocytic parasite is transmitted primarily by *Ixodes ricinus* ticks and also has the characteristics necessary to be transmitted by blood transfusion [4, 5].

Symptomatic infection caused by *B. divergens* is relatively rare in humans. When occurring, it causes fever, anemia, jaundice, hemoglobinuria and renal failure. Sometimes it progresses to a fulminant disease with high mortality, mainly in immunocompromised and elderly patients, especially in those with asplenia [2].

In contrast to the low incidence of full-blown clinical babesiosis in Europe, the number of persons with *Babesia* antibodies that are asymptomatic or paucisymptomatic is significant. *Babesia* IgG prevalence ranges from 2% to 39.7% in different populations [6–10], suggesting that most of the *Babesia* infections remain undetected and may be more frequent than suspected [2, 11, 12]. These undetected infections could also represent a potential risk for blood donation safety in European regions with evidence of human babesiosis [2, 4, 5, 9, 13].

Human babesiosis is considered very uncommon in Spain, but recent works have demonstrated that its incidence in hospitalized patients has increased from 1997 through 2019 (0.28 cases per 10,000,000 population-year). In addition, it entails an elevated hospital cost per patient (6445.71 euros) and an associated mortality rate of around 6.9% (2 deaths/29 total cases of babesiosis, although both dead patients had significant comorbidities) [14, 15].

Asturias, which is located along the North Atlantic coast in Northwestern Spain, is the region where challenging human babesiosis cases have occurred in recent years [16, 17]. Asturias has a total area of 10,603.57 km^2^ divided into 78 councils. It is one of the largest forest areas in Spain and constitutes the perfect environment for tick-borne diseases, including endemic Lyme disease caused by *Borrelia burgdorferi* sensu lato and human babesiosis, whose incidence among hospitalized patients was 0.41 cases per 10,000,000 population-year from 1997 through 2019 [14, 18–21].

One of the human babesiosis cases reported in Asturias was an exceptionally severe infection caused by *B. divergens* in a immunocompetent, healthy, 46-year-old forest ranger [16], whose clinical manifestations did not match the “classic description” of European babesiosis [1]. This case and two other cases of young (35 and 37 years old), immunocompetent individuals with normal spleen sizes from France [22] suggest that they had an unknown susceptibility to this infection. Another severe case corresponded to an 87-year-old immunocompromised Asturian woman with intact spleen who died of babesiosis [17].

These two Asturian cases raised concerns that underlying human babesiosis might be occurring in Asturias. Therefore, we retrospectively evaluated the seroprevalence of human babesiosis in 74 Asturian councils from 2015 through 2017, a period covering the intermediate years in which these severe human babesiosis cases occurred.

Since *Ixodes ricinus* is the tick species that transmits babesiosis and Lyme disease, we examined serum samples from patients that were positive for *B. burgdorferi* s.l. IgG, a condition that indicates previous exposure to tick bites [8]. Next, we analyzed and compared the epidemiological and serological factors among patients infected solely with *B. burgdorferi* s.l. and those who also had serological evidence of *B. divergens* infection and discussed the epidemiological importance of human babesiosis in Asturias.

## Methods

### Study design and populations

This study included 120 *B. burgdorferi* s.l. IgG-positive patients who resided, from 2015 through 2017, in 74 out of 78 councils of Asturias (Northwestern Spain). Patients from four Asturias coastal councils were excluded from the study because their *Borrelia* serology tests were done in their reference hospitals, different from the Hospital Universitario Central de Asturias (HUCA), Oviedo.

According to the Spanish National Statistical Office (https://www.ine.es), the 74 councils, which occupy a surface area of 9997 km^2^, were inhabited by 658,186 residents in 2017. The population density of these councils was 65.84 inhabitants/km^2^ and represented 66.3% of the total population and 94.3% of the total Asturian territory.

The patients in this study were admitted to or were externally followed at the Infectious Diseases, Neurology, Rheumatology or Dermatology Services of the HUCA or its regional affiliated hospitals.

Patients’ serum samples were collected, tested for Lyme disease and kept at – 80 °C at the Microbiology Service of the HUCA. Detection of *B. burgdorferi* s.l. IgG antibodies was done by using an automated qualitative test (Vidas, BioMerieux, Madrid, Spain). Results were confirmed by immunoblot (*Borrelia* IgG IgM EcoLine, Sekisui Diagnostics, Rüsselsheim, Germany) [21]. This study also included three positive serum controls as follows: from the Asturian elderly patient who died of *B. divergens* babesiosis [17], from a Spanish patient from outside Asturias co-infected with HIV and *Babesia divergens*, who suffered a severe babesiosis that was diagnosed by our group, and from a Finnish patient doubly infected with *B. burgdorferi* s.l. and *B. divergens* who died of babesiosis [23, 24]. A human sera pool positive for *B. microti*, a sera pool positive for malaria and a human sera pool negative for different infectious diseases, including toxoplasmosis, malaria and babesiosis, that have been tested in previous studies [16, 17, 23, 25, 26], were used as negative controls. To detect *B. divergens* IgG antibodies in the samples seropositive for *B. burgdorferi* s.l., we used our in-house indirect fluorescent assay (IFA) [16, 17, 23] with slight modifications. We developed a novel Western blot (WB) by using *B. divergens* (Rouen 87 strain) protein extracts as targets to evaluate the humoral antibody response in the patients infected with *B. divergens*. We also utilized our previous WB based on the use of a major surface protein of *B. divergens* (Bd37) as a target [23, 27] with slight modifications.

### Detection of *B. divergens* IgG antibodies by indirect fluorescent assay

To carry out our in-house IFA, 1 × 10^7^ cells/ml from *B. divergens* (Rouen 87 strain) infected human erythrocyte cultures (25%–30% parasitemia) [28, 29], were pipetted onto 16-wells slides (10 μl of culture per well) (Thermo Scientific, Portsmouth, NH, USA) and incubated at 37 °C for 30 min. Then, the slides were fixed in cold 50% acetone–50% methanol, washed three times in phosphate-buffered saline (PBS) and incubated at 37 °C for 1 h with *B. burgdorferi* s.l. seropositive samples and controls. All serum samples and controls were tested in triplicate. According to World Health Organization (WHO) guidelines, the cut-off titers used for discrimination between seronegative and seropositive reactions by IFA were set at 1:128 for *B. divergens* [6]. Bound antibody was detected by using fluorescein isothiocyanate-conjugated anti-human IgG diluted 1:200 in PBS (Invitrogen, Eugene, OR, USA). The preparations were counterstained with 5 μl (2.5 μg/ml in PBS) of DAPI (Pierce, Rockford, IL, USA) per well and examined by fluorescence microscopy (Leica DH 2000 LED, Wetzlar, Germany).

### Detection of *B. divergens* IgG antibodies by Western blot

*Babesia divergens* (Rouen 87 strain)-infected and non-infected human erythrocytes from in vitro cultures (30%–40% parasitemia) [28, 29] were solubilized into three volumes of saponin buffer (0.15% of saponin in PBS) and incubated at 37 °C for 20 min. Then, samples were washed by centrifugation with PBS. Pellets were resuspended in PBS with a protease inhibitor mixture (Sigma-Aldrich, St. Louis, MO, USA) and 2 × Laemmli sample buffer (National Diagnostics, Atlanta, GA, USA) before electrophoresis. To produce the Bd37 recombinant protein (GST-rBd37), *B. divergens* DNA encoding Asn^28^ to Phe^341^ of Bd37 [27] was amplified by PCR and cloned into the expression plasmid vector pGEX-4T1 (GE Healthcare, Uppsala, Sweden) according to the manufacturer's instructions. *Escherichia coli* strain BL21-gold (DE3) plysS (Agilent Technologies, Santa Clara, CA, USA) was transformed with the Bd37 expression plasmid. Then, super optimal broth (SOB) medium (250 ml) containing 100 μg/ml ampicillin (Sigma-Aldrich) was inoculated with 1 ml of fresh overnight transformed *E. coli* strain BL21-gold cultured and grown at 37 °C to A_600_ = 0.6 before induction with 0.5 mM isopropyl-β-d-thiogalactopyranoside (IPTG), (Sigma-Aldrich). After 3 h of induction to produce the GST-rBd37, cells were pelleted and resuspended in B-PER bacterial protein extraction reagent (Thermo Scientific) supplemented with a protease inhibitor mixture (Sigma-Aldrich) for 10 min. Then, the insoluble material was removed by centrifugation, and the soluble fraction was used to purify the GST-rBd37 by using glutathione-Sepharose 4B (GE Healthcare) as described by the manufacturer. The glutathione-S-transferase (GST) fusion partner (26 kDa) for Bd37 was also produced and purified following the same procedure.

*Babesia divergens* protein extracts and the GST-rBd37 were used as target substrates for WB. Non-parasitized human erythrocytes protein extracts and GST recombinant protein were also used as controls. Thirty μg of protein extracts and 150 ng of recombinant GST-rBd37 and GST proteins were loaded per well on 12.5% SDS-PAGE gel and transferred to Amersham Protran nitrocellulose membranes (GE Healthcare) at 80 mA for 1 h at room temperature. Membranes were blocked in a blocking solution made of PBS with 0.05% Tween-20 (PBS-T) and 3% (w/v) bovine serum albumin (Sigma-Aldrich) and incubated for 1 h at room temperature with patients’ serum samples and controls diluted 1:128 in the blocking solution. Then, membranes were treated with horseradish peroxidase-conjugated anti-human IgG (1:10,000 dilution) (Thermo Scientific) for 1 h at room temperature and washed with washing buffer (PBS + 0.05% Tween-20). Antigen detection was assessed by a colorimetric reaction (CN-DAB Substrate kit, Thermo Scientific).

### Statistical analysis

Diverse epidemiological and risk factors were evaluated and compared in patients infected with *B. burgdorferi* s.l. and with IgG antibodies against *B. divergens* versus those infected only with *B. burgdorferi* s.l. The non-parametric Mann-Whitney *U* test was used to assess the differences in age, whereas the Chi-square test and Fisher’s exact text, when appropriate, were used to compare proportions between the two study groups. *Borrelia burgdorferi* s.l.-*B. divergens* incidences during the study period were calculated for each council where *B. divergens* IgG antibodies were detected as well as for the 74 councils included in the study as a whole. The SPSS software v. 25 (IBM Corp., Armonk, NY, USA) was used for statistical calculations. The limit for statistical significance was established at the < 0.05 level for a two-sided test.

## Results

**Fig. 1:**
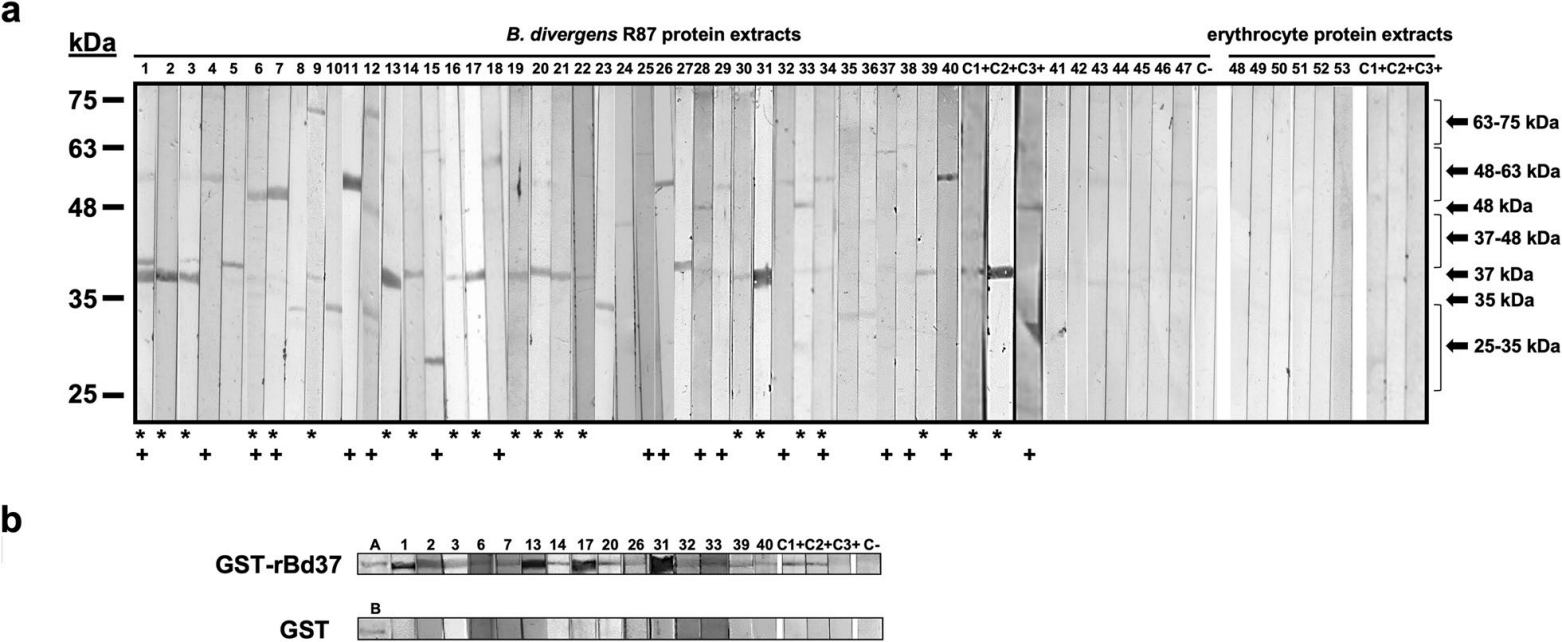
Serological detection of specific anti-*B. divergens* IgG antibodies by Western blot (WB). Panel **a**: WB was prepared by using *B. divergens* (Rouen 87) and non-infected human erythrocyte protein extracts. Blots were incubated with sera of patients infected with *B. burgdorferi* s.l. and positive and negative controls. Lanes 1–40: different *B. divergens* proteins, ranging from 25 to 75 kDa, that were identified from serum patient samples. Protein bands differed in number, frequency, size and intensity between patients. Lanes 41–47: some of the serum samples from patients infected with *B. burgdorferi* s.l. that did not recognize *B. divergens* proteins. Lanes 48–53 and lines C1+, C2+ and C3+: Some of the serum samples and positive controls that did not recognize erythrocyte proteins. Serum samples that reacted against a native *B. divergens* protein with ~ 37 kDa (*). Serum samples that reacted against native *B. divergens* proteins of ~ 48–63 kDa (+). Panel **b**: WB was prepared by using the recombinant GST-rBd37 (Rouen 87) protein and the GST fusion partner for Bd37. Specificity from the patients’ sera against recombinant Bd37 from *B. divergens* was observed by WB. Lanes A and B: GST-rBd37 (59.5 kDa) and GST (26 kDa) recombinant proteins, respectively, subjected to SDS-PAGE. The number of the lanes indicates which serum sample from Panel a recognized the GST-rBd37 protein. Positive control 1 (C1+): serum sample from an Asturian patient [23]. Positive control 2 (C2+): serum sample from a non-Asturian Spanish patient co-infected with HIV and *B. divergens* [25]. Positive control 3 (C3+): serum sample from a Finnish patient doubly infected with *B. burgdorferi* s.l and *B. divergens*. Negative control (C−) consisted of a human sera pool negative for different infectious diseases, including toxoplasmosis, malaria and babesiosis. None of the sera reacted with the recombinant GST protein

*Babesia divergens* IgG antibodies were detected by IFA in 39.2% (47/120) of the serum samples from patients infected with *B. burgdorferi* s.l. Similar result was observed by WB using *B. divergens* protein extracts, given that 40 serum samples of the total were positive by this technique (33.3%). Serum samples identified different *Babesia divergens* protein profiles ranging in size from 25 to 75 kDa. The most frequent IgG antibody response was against a ≈37-kDa protein that was recognized by 47.5% (*n* = 19) of the serum samples, followed by two larger proteins ranging between ≈48 and 63 kDa that were recognized by 45% (*n* = 18) of them (Table 1; Fig. 1).

**Table 1:** Results from patients with positive *Babesia divergens* antibodies detected by indirect fluorescent assay (IFA) and Western blot

Sample (*n*)	IFA	Western blot	
	*B. divergens* IgG antibodies (1:128)	*B. divergens* protein extracts IgG antibodies (1:128)	GST-rBd37*IgG antibodies (1:128)
1	+	+	+
2	+	+	+
3	+	+	+
4	+	+	−
5	+	+	−
6	+	+	+
7	+	+	+
8	+	+	−
9	+	+	−
10	+	+	−
11	+	+	−
12	+	+	−
13	+	+	+
14	+	+	+
15	+	+	−
16	+	+	−
17	+	+	+
18	+	+	−
19	+	+	−
20	+	+	+
21	+	+	−
22	+	+	−
23	+	+	−
24	+	+	−
25	+	+	−
26	+	+	+
27	+	+	−
28	+	+	−
29	+	+	−
30	+	+	−
31	+	+	+
32	+	+	+
33	+	+	+
34	+	+	−
35	+	+	−
36	+	+	−
37	+	+	−
38	+	+	−
39	+	+	+
40	+	+	+
41	+	−	−
42	+	−	−
43	+	−	−
44	+	−	−
45	+	−	−
46	+	−	−
47	+	−	−

^*^GST-rBd37recombinant protein

The *B. divergens* ≈37 kDa protein could correspond in size with the major surface protein of *B. divergens*, Bd37, which is also 37 kDa [27]. To assess this hypothesis we used the corresponding recombinant GST-rBd37 protein as a substrate to detect specific Bd37 IgG antibodies in the positive serum samples by WB.

Of the 40 serum samples tested, 12 samples recognized both the ≈37 kDa protein present in parasite protein extracts and also the GST-rBd37 protein. Moreover, three serum samples that did not initially show a ≈37 kDa protein in their protein profile latterly responded against the GST-rBd37 protein (Table 1; Fig. 1). Antibodies against the ≈37 kDa and GST-rBd37 proteins were also present in two of the three positive control sera [17, 23], while the third one, from a patient infected with both *B. burgdorferi* s.l and *B. divergens* [24], recognized one of the ≈48–63-kDa proteins (Fig. 1). Cross-reactivity with negative controls or against erythrocyte protein extracts and GST was not observed.

Western blot confirmed 85.1% of the positive serum samples detected by IFA. Moreover, 32% of the serum samples presented specific antibodies against the Bd37 protein.

According to IFA, the incidence of babesiosis in patients who were attended at the HUCA and regional affiliated hospitals during the study period was 7.14 per 100,000 population. There were no significant differences between monoinfected patients with *B. burgdorferi* s.l. and patients infected with *B. burgdorferi* s.l. and with *B. divergens* IgG antibodies in the diverse epidemiological and risk factors studied (Table 2). The median age of the patients with *B. burgdorferi* s.l. infection and detectable *B. divergens* antibodies was 56.4 years (IQ range 45.5–71.8). These patients had a mild clinical course, including two patients who had a known immunodepression. Most (82.9%, *n* = 34) developed tick-bite risky outdoor activities and some had non-professional contact with animals (39.0%, *n* = 16). However, only nine of these patients worked in agricultural, livestock or fishery sectors (Table 2). All patients infected with *B. burgdorferi* s.l. and with *B. divergens* IgG antibodies lived in 22 of the 74 Asturian councils under study. A third of the total population studied resided in Oviedo, an urban council inhabited by 220.300 people where 11 patients had *B. divergens* IgG antibodies. The rest resided in rural councils surrounded by Nature Reserves.

**Table 2:** Demographic and epidemiological characteristics and predisposing factors of patients solely infected with *Borrelia burgdorferi* s.l. and patients infected with *B. burgdorferi* s.l. who also had *Babesia divergens* IgG antibodies

	All (*n* = 120)	*B. burgdorferi s.l.* (*n* = 73)	*B. burgdorferi s.l./B.divergens* (*n* = 47)	Statistics
Demography and epidemiology				
Gender				
M	81 (67.5%)	47 (64.4%)	34 (72.3%)	*χ*^2^ = 0.825, *df* = 1, *P* = 0.364
F	39 (32.5%)	26 (35.6%)	13 (27.7%)	
Age				
Years (n = 119)	57.19 (43.50–72.24)	58.0 (42.8–73.6)	56.4 (45.5–71.8)	*U* = 1649.4, *Z* = -0.231, *P* = 0.817
Occupation				
Farmer	8(10.0%)	4 (8.5%)	4 (12.1%)	*χ*^2^ = 4.90, *df* = 3, *P* = 0.179
Cattle breeder	5 (6.3%)	3 (6.4%)	2 (6.1%)	
Open air activity	3 (3.8%)	0 (0%)	3 (9.1%)	
Other	64 (80.0%)	40 (85.1%)	24 (72.7)	
Tick bite identified				
Yes	26 (23.2%)	17 (24.6%)	9 (20.9%)	*χ*^2^ = 0.204, *df* = 1, *P* = 0.651
No	86 (76.8%)	52 (75.4%)	34 (79.1%)	
Tick removal				
Yes	20 (17.9%)	12 (17.4%)	8 (18.6%)	*χ*^2^ = 0.027, *df* = 1, *P* = 0.87
No	92 (82.1%)	57 (82.6%)	35 (81.4%)	
Predisposing factors				
Yes	106 (91.4%)	64 (90.4%)	42 (93.3%)	*χ*^2^ = 0.356, *df* = 1, *P* = 0.738
No	10 (8.6%)	7 (9.6%)	3 (6.7%)	
Transfusion				
Yes	0 (0%)	0 (0%)	0 (0%)	-
No	114 (100%)	69 (100%)	45 (100%)	
Asplenia				
Yes	0 (0%)	0 (0%)	0 (0%)	-
No	114 (100%)	69 (100%)	45 (100%)	
Immunosuppression				
Yes	4 (3.5%)	2 (2.9%)	2 (4.4%)	*χ*^2^ = ,0.206 *df* = 1, *P* = 0.644
No	111 (96.5%)	68 (97.1%)	43 (95.6%)	
Age > 50 years				
Yes	73 (63.9.6%)	43 (59.7%)	33 (70.2%)	*χ*^2^ = 1.356, *df* = 1, *P* = 0.244
No	43 (36.1%)	29 (40.3%)	14 (29.8%)	
Outdoor hobbies				
Yes	79 (76.7%)	45 (72.6%)	34 (82.9%)	*χ*^2^ = 1.478, *df* = 1, *P* = 0.224
No	24 (23.3%)	17 (27.4%)	7 (17.1%)	
Non-professional animal contact				
Yes	34 (33.0%)	18 (29.0%)	16 (39.0%)	*χ*^2^ = 1.114, *df* = 1, *P* = 0.291
No	69 (67.0%)	44 (71.0%)	25 (61.0%)	

*Age is described as median (IQ range) and the remainder variables as n (%)

**Fig. 2:**
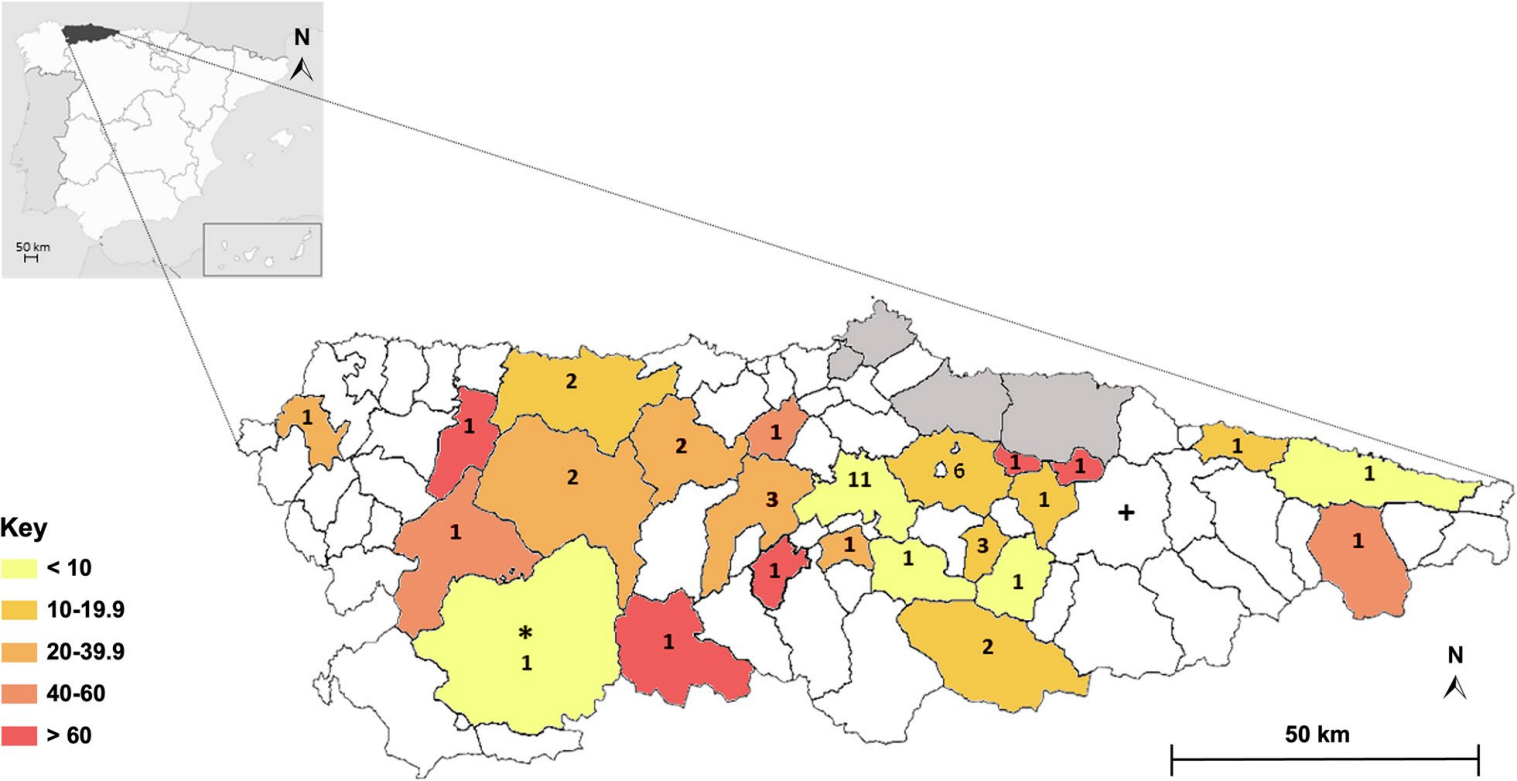
Geographical distribution of patients who had antibodies against *B. divergens*. Map shows the location of Asturias in Spain and the Asturian councils where *B. divergens* infected patients lived from 2015 through 2017. The number of infected patients appears associated to each council. The map also shows the councils where severe (*) and fatal (+) human babesiosis cases occurred in Asturias [16, 17]. The color blocks represent the range of incidence of babesiosis per 100,000 population in each council

Of note, 66% of the patients with babesiosis were centripetally grouped in 20 contiguous councils of Central Asturias, a large area that also includes the councils where the two severe human babesiosis cases were reported [16, 17] (Fig. 2).

## Discussion

From 2015 through 2017, at least 47 Asturian patients had serological evidence of both *B. burgdorferi* s.l. and *B. divergens* infections, although our study cannot determine whether both infections were simultaneous or sequential.

The seroprevalence of *B. divergens* antibodies in our patients infected with *B. burgdorferi* s.l. (39.2%) was similar to the 33.2% reported in 199 Belgian patients with history of tick bites and tick-borne disease symptoms [7] but higher than the 16.3% seroprevalence rate of *B. microti-B. divergens* antibodies reported in 86 Swedish individuals infected with *B. burgdorferi* s.l. [8].

Despite differences in local seroprevalence rates, all reports point out that persons with *Babesia*-positive antibodies exceed the number of clinically diagnosed human babesiosis cases from these, and even from other European geographical areas [1, 2]. However, as shown in this retrospective study, human babesiosis passes unnoticed by health systems, local blood transfusion services and surveillance plans. A low index of suspicion, asymptomatic babesiosis and confusion with common viral-like illnesses or with Lyme disease could mask babesiosis and might explain discrepancies between high seroprevalence and low clinical incidence.

Indeed, the babesiosis incidence that we observed (7.14 cases per 100,000 population) does not represent the true incidence of the infection. It is expected to be much higher, but it was biased low because we studied *B. divergens* infection incidence only in those symptomatic patients with Lyme disease who searched for care at the HUCA and HUCA-affiliated secondary hospitals.

Contrarily, it is very unlikely that other undiagnosed symptomatic babesiosis cases were overlooked for several reasons. First, the territory studied represented almost 95% of the total surface of Asturias. This area is sanitarily covered by the HUCA and its affiliated regional hospitals and all serological determinations are centralized in the microbiology laboratory of the HUCA. Second, the HUCA is the tertiary referral hospital of Asturias. Thus, complicated and unusual cases such as those of severe babesiosis are transferred to the HUCA for hospital care.

This study failed to find significant differences in the diverse epidemiological and risk factors analyzed between patients infected with *B. burgdorferi* s.l. and those infected with *B. burgdorferi* s.l. who also had *B. divergens* IgG antibodies. Our work suggests that the epidemiology of babesiosis is equally shared by the two infections. However, the relatively small sample size of this study and its retrospective nature may have overlooked minor differences between the two groups.

At this time, 40 doubly infected patients showed different patterns of antibody response against *B. divergens* by WB, the recognition of Bd37 present in *B. divergens* (Rouen 87) protein extracts prevailing, and in the form of recombinant protein (GST-rBd37 Rouen 87). *Babesia divergens* 48–63-kDa antigens were also dominant.

Notably, not all WB-positive serum samples had specific antibodies against the Bd37 major surface antigen. Discrepancies in the recognition of Bd37 in *B. divergens* protein extracts, but not of recombinant protein, were also observed in seven patients. The large genetic diversity of the *B. divergens* strains infecting humans [2], the polymorphism within the Bd37 protein between strains [30] and even the individual humoral immune response of each patient could at least partly explain these discrepancies, especially as only the *B. divergens* Rouen 87 strain was used for WB.

Interesting, three serum samples that did not recognized Bd37 in *B. divergens* protein extracts were able to recognize the recombinant GST-rBd37 protein. The quantity, quality and purity of the recombinant GST-rBd37 protein used in the WB compared to *Babesia divergens* protein extracts may favor the detection of specific IgG antibodies against Bd37, especially when antibody levels in patients’ sera are low.

The WB system was also unable to determine the humoral response in seven of the IFA-positive patients. As discussed above, the genetic diversity of *B. divergens* strains may explain the negative WB results and suggests that it may be convenient to use different *B. divergens* strains whenever possible to improve serological results in terms of sensitivity and specificity.

Although B lymphocytes and their production of antibodies are particularly important for the eradication of the babesiosis infection [31], there is still little information about the humoral immune responses elicited in patients with different grades of babesiosis severity.

To our knowledge, the humoral response against *B. divergens* has been analyzed in few patients. The chronological humoral response of two patients consisted in a constant response against a 36-kDa antigen from the first days post-hospitalization onwards and progressive response against other antigens, including a protein of 37 kDa, in the following days to months after the *B. divergens* diagnosis [28]. *Babesia divergens* proteins of 33 and 37 kDa were also detected by WB using serum samples from two breeders with a history of tick bite [32]. A study on the humoral response to *B. divergens* also revealed *B. divergens* IgG antibodies against the recombinant Bd37 protein in the serum of our HIV-infected patient [23].

However, it is still unclear whether these specific antibody repertories are similar or different among patients and whether they could modulate the efficacy of the humoral protective immune response and the disease outcome in patients monoinfected with *B. divergens* and in those concomitantly or serially infected with *B. burgdorferi* s.l.

As for infections caused by other *Babesia* species [33, 34], additional studies are needed to evaluate the humoral immune response during mono- and double infections with *B. divergens* and *B. burgdorferi* s.l. and to identify antigens that elicit the production of protective antibodies or to determine whether a supposedly healthy blood donor has an asymptomatic infection or a patient has an acute, chronic or past babesiosis.

Limitations of this study include those related to retrospective studies, including the gathering of data from old medical records. To our knowledge, the sample size of our study was the largest published so far for *B*. *burgdorferi* s.l. and *B. divergens* double infections. However, it may not have been large enough to detect subtle statistical differences between the two comparison groups resulting in a type II error. Also, the true incidence of the infection was surely underestimated in our study because not all babesiosis asymptomatic or paucisymptomatic persons do seek medical care. However, the centralization of the serological studies in a single laboratory ensures that no patient in which these infections were suspected was overlooked.

Similar scenarios could be occurring in other areas of Spain [14] and in other European regions that are also affected by human babesiosis [2] and where seroprevalence studies would be desirable.

## Conclusions

This study evaluated the seroprevalence, epidemiology and risk factors of human babesiosis in patients seropositive for Lyme disease and determined the location of *B. divergens* in a specific period in Asturias.

The high babesiosis seroprevalence detected in patients infected with *B. burgdorferi* s.l. reveals that *B. divergens* could have circulated for several years in Asturias and may pose a threat to human health.

No differences were observed among the diverse epidemiological and risk factors analyzed between patients monoinfected with *B. burgdorferi* s.l. and those who also had serological evidence of infection with *B. divergens*.

However, patients showed different antibody response patterns against *B. divergens*. This result could be relevant to the host defense and lays the bases for future studies directed towards characterizing the individual humoral response against specific *B. divergens* antigens.

Since Asturias meets the natural conditions for the transmission of *Babesia*, the appearance of new babesiosis cases ranging from asymptomatic to severe infections are expected in the coming years. Moreover, there is clear evidence that the Asturian population has been exposed to *B. divergens*, which could entail some risks for the community health and for transfusion-transmitted babesiosis.

This local epidemiological evidence of babesiosis makes Asturias an emerging risk area for this zoonosis. Human babesiosis could also be relevant in other Spanish and European regions. Therefore, health surveillance plans might be desirable to evaluate tick-borne diseases seroprevalence, early diagnosis and blood screening and to develop control strategies in the future.

## Data Availability

All data generated or analyzed during this study are included in this published article.

## Abbreviations

Bd37: Major surface protein of *B. divergens*
DAPI: 4′,6-Diamidino-2-fenilindol
g: Gram
*χ*^2^: Chi-square test
*df*: Degrees of freedom
GST: Glutathione-*S*-transferase
GST-rBd37: Bd37 recombinant protein
HIV: Human immunodeficiency virus
h: Hour
HUCA: Hospital Universitario Central de Asturias
IFA: Indirect fluorescent assay
IgG: Immunoglobulin G
IPTG: Isopropyl-β-d-thiogalactopyranoside
kDa: Kilodalton
km^2^: Square kilometer
mA: Milliampere
µg: Microgram
*P*: *P-value*
PBS: Phosphate-buffered saline
PBS-T: PBS with 0.05% Tween-20
SOB: Super optimal broth
SDS-PAGE: Sodium dodecyl sulfate-polyacrylamide gel electrophoresis
*U*: Mann–Whitney *U* test
WB: Western blot
WHO: World Health Organization
